# Internet-Based Supportive Interventions for Family Caregivers of People With Dementia: Randomized Controlled Trial

**DOI:** 10.2196/50847

**Published:** 2024-10-04

**Authors:** Yanhong Xie, Shanshan Shen, Caixia Liu, Hong Hong, Huilan Guan, Jingmei Zhang, Wanqi Yu

**Affiliations:** 1 Geriatrics Department, Zhejiang Hospital, Hangzhou, China; 2 Nursing Department, Zhejiang Hospital, Hangzhou, China; 3 General Surgery, Zhejiang Hospital, Hangzhou, Zhejiang Province, China; 4 The Medical Record Statistics Department, Zhejiang Hospital, Hangzhou, China

**Keywords:** dementia, family caregiver, web-based training, care burden, care ability, caregivers, carer, caregiving, informal care, RCT, controlled trial, randomized, gerontology, geriatric, older adult, elder, elderly, older person, older people, ageing, aging, dementia care, randomized controlled trial, internet-based training

## Abstract

**Background:**

As dementia progresses, patients exhibit various psychological and behavioral symptoms, imposing a significant burden on families and society, including behavioral and psychological symptoms of dementia. However, caregivers lack professional care knowledge and skills, making it difficult for them to effectively cope with the diverse challenges of caregiving. Therefore, it is necessary to provide caregivers with professional knowledge and skills guidance.

**Objective:**

This study aimed to analyze the impact of internet-based training on behavioral and psychological symptoms of dementia in patients, and explore how this training model affects the caregiving abilities and caregiving burden of the family caregivers of patients with dementia.

**Methods:**

Using a consecutive enrollment method, the Department of Geriatrics at Zhejiang Hospital (Zhejiang, China) recruited 72 informal caregivers of patients with dementia. These caregivers were randomly divided into an intervention group and a control group, with 36 participants in each group. The intervention group underwent caregiver skill training via a web-based platform, whereas the control group initially received face-to-face follow-up guidance and was subsequently offered web-based training after 6 months. To assess the effectiveness of the intervention program, we used the Neuropsychiatric Inventory Questionnaire (NPI-Q), the Chinese version of the Zarit Burden Interview (CZBI), and the Sense of Competence in Dementia Care Staff Scale (SCIDS) for evaluations conducted before the intervention, 3 months after the intervention, and 6 months after the intervention.

**Results:**

Between July 2019 and December 2020, a total of 66 patients successfully completed the intervention and follow-up. After 6 months of intervention, the NPI-Q score of the intervention group was 3.18 (SD 3.81), the CZBI score was 10.97 (SD 5.43), and the SCIDS score was 71.88 (SD 4.78). The NPI-Q score of the control group was 8.09 (SD 8.52), the CZBI score was 30.30 (SD 13.05), and the SCIDS score was 50.12 (SD 9.10). There were statistically significant differences in NPI-Q (*P*=.004), CZBI (*P*<.001), and SCIDS scores (*P*<.001) between the intervention group and the control group. Repeated measures analysis of variance showed that compared with before the intervention, there were statistically significant differences in CZBI (*P*<.001) and SCIDS (*P*<.001) scores 3 months after the intervention, while the difference in NPI-Q (*P*=.11) scores was not significant. The total scores of NPI-Q (*P*<.001), CZBI (*P*<.001), and SCIDS (*P*<.001) were significantly improved 6 months after the intervention. In addition, the results of the covariance analysis showed that after excluding the time effect, the web-based training intervention significantly reduced the NPI-Q score (−2.79, 95% CI −4.38 to −1.19; *P*<.001) of patients with dementia and the CZBI score (−13.52, 95% CI −15.87 to −11.16; *P*<.001) of caregivers, while increasing the SCIDS score (12.24, 95% CI 9.02-15.47; *P*<.001).

**Conclusions:**

Internet-based training could significantly reduce the level of behavioral symptoms in older patients with dementia and alleviate the burden on caregivers, enhancing their caregiving abilities. Our results confirmed the effectiveness and feasibility of web-based training, which was of great significance in providing caregiving knowledge training for informal caregivers of persons with dementia.

## Introduction

Dementia care remains a major public health challenge for global health systems. According to a recent nationwide cross-sectional study [1], 15.07 million individuals older than 60 years experienced dementia in China. A person with dementia, particularly one with moderate-to-severe dementia, has extensive health and social care needs. The annual total treatment costs of patients with Alzheimer disease in China are predicted to reach US $507.49 billion in 2030 and US $1.89 trillion in 2050 [2]. Dementia severity is an important driver of cost and the proportion of mild, moderate, and severe dementia cases living in a country can influence the cost estimates. In China, 70% of patients with dementia live at home and are cared for by their spouses, children, or other relatives. According to a survey conducted by Alzheimer Disease Chinese [3], there are 3 major difficulties faced by the families of patients with dementia, which mainly are insufficient care capacity, a lack of care resources, and single treatment services. According to the research report [3], 65.43% of caregivers have no hope of treatment and feel intense psychological pressure, 68.69% of caregivers report that their health has been affected, and 78.39% of caregivers said that their social life is often affected.

Behavioral and psychological symptoms of dementia (BPSD) may occur at any stage of the disease progression, with patients exhibiting at least one type of BPSD. BPSD is highly correlated with caregiver burden. Caregiver factors are some of the causes and triggers of BPSD, and insufficient interaction between caregivers and care recipients may lead to the occurrence or exacerbation of BPSD. For family caregivers, caring for people with dementia is perceived as one of the most stressful experiences. During the long-term progression of the disease, the patient’s independence will decline, which requires more responsibility and supervision time from informal caregivers. Long-term problems of continuously managing activities of daily living [4,5], behavioral and psychological symptoms [6], and providing emotional, spiritual, and social support place considerable burdens on the family, which can result in mental health problems such as depression. The burden of care can put caregivers at risk of physical and psychological ailments, as well as negatively affect their quality of life, so effective and practical support is essential. Training and supporting family caregivers, especially in the proper management of BPSD, to enhance their ability to cope with BPSD, may help break this vicious cycle [7].

In 2019, informal dementia caregivers spent over 89 billion hours providing support with activities of daily living about 5 hours per day per person with dementia [8]. As the most direct contact and participant, the competence of the caregiver has a direct impact on the outcome of BPSD. However, due to the ongoing COVID-19 pandemic, many support services for dementia caregivers have been reduced, delayed, or even withdrawn, which has substantially increased interest in web-based health services [9]. It is important to create a digital platform that offers invaluable and usable information to caregivers of people living with dementia [10]. In the face of these significant challenges, the WHO developed “iSupport,” an evidence-based e-health intervention designed to help dementia caregivers provide good care and take care of themselves [11]. Internet-based supportive interventions can provide convenient and efficient support and education to potentially reduce the physical and psychological burden associated with providing care. Internet-based supportive interventions for family caregivers of people with dementia have been reported in the United Kingdom [11], India [12], the Netherlands [13], Brazil [14], Germany [15], and other countries, but they are rare in China.

This manuscript presents the findings of a randomized controlled trial examining the implementation of internet-based supportive interventions for home care among patients with dementia in China. The trial is an innovative web-based support program that was developed to advance the skills, knowledge, and practice of caregivers, in order to enhance self-care skills in individuals with dementia and simultaneously provide invaluable assistance to their caregivers. The experiment also aims to assess the effectiveness of the web-based support program, and to explore its impact on the caregiving burden and caregiving abilities of dementia caregivers, compared with dementia caregivers who receive routine caregiving interventions after discharge. The primary outcomes of this study consisted of evaluating the severity of neuropsychiatric symptoms in patients with dementia, assessing the burden on caregivers, and gauging the capability of caregivers. We hypothesized that internet-based supportive interventions would lead to greater improvement in primary outcomes than routine caregiving interventions after discharge.

## Methods

### Study Design

The trial was designed, planned, and executed by the Department of Geriatrics of Zhejiang Hospital (Zhejiang China). A randomized controlled trial design was used to study the feasibility and effectiveness of a nurse-led multidisciplinary team web-based training and support program. A total of 72 caregivers of patients with dementia were recruited from the Geriatric Department of Zhejiang Hospital from July 2019 to December 2020. The recruitment strategy included posting flyers and posters in the geriatric ward. During the patient’s hospitalization, the geriatric nurse proposed this protocol to the family caregivers of the patient with dementia. Interested participants were provided with a flyer containing contact information for the research and filled out a contact form. The geriatric nurse confirmed the inclusion criteria and collected the signed informed consent. The control group intervention measures included regular face-to-face follow-up interviews with caregivers to provide education on dementia care knowledge and skills. These measures were conducted every 3 months after the patient’s discharge. All participants were assessed at baseline (T0) and postintervention (T1, 3 months after T0; T2, 6 months after T0). Once enrolled, participants had access to the web-based program or follow-up interview for more than 6 months.

### Ethical Considerations

All procedures were in accordance with the Helsinki Declaration, and the study was approved by the Ethics Review Committee of Zhejiang Hospital (2019 pretrial case number: 23K). It was registered at the Chinese Clinical Trial Registry (ChiCTR-2200057858). A written informed consent form was sought from each participant.

### Inclusion and Exclusion Criteria

Selection criteria included the following: (1) being a primary, informal caregiver aged at least 18 years; (2) caring for individuals with dementia while living together at home for a minimum of 6 months; (3) having internet access via computers or iPads; and (4) could read, understand Chinese, and following instructions. Participants were excluded if they had severe visual or hearing impairment that was incompatible with participation as assessed by the study staff.

### Recruitment, Consent, and Baseline Data Collection

Between July 2019 and December 2020, 72 informal primary caregivers of people with dementia of all subtypes and stages were recruited via the Department of Geriatrics, Zhejiang Hospital. Caregivers meeting the eligibility criteria were informed of initial details about the study and provided with a subject information sheet. Once potential participants decided to participate, they were asked to sign the informed consent form and were informed of their rights. Baseline information was collected by trained interviewers from participants through the completing standardized questionnaires. During the interviews, trained interviewers were blinded to the group allocations. Participants were randomly allocated on a 1:1 ratio assigned to either the intervention or a waiting list control group after baseline assessment. Randomization was carried out using a random number generator.

**Figure 1.**
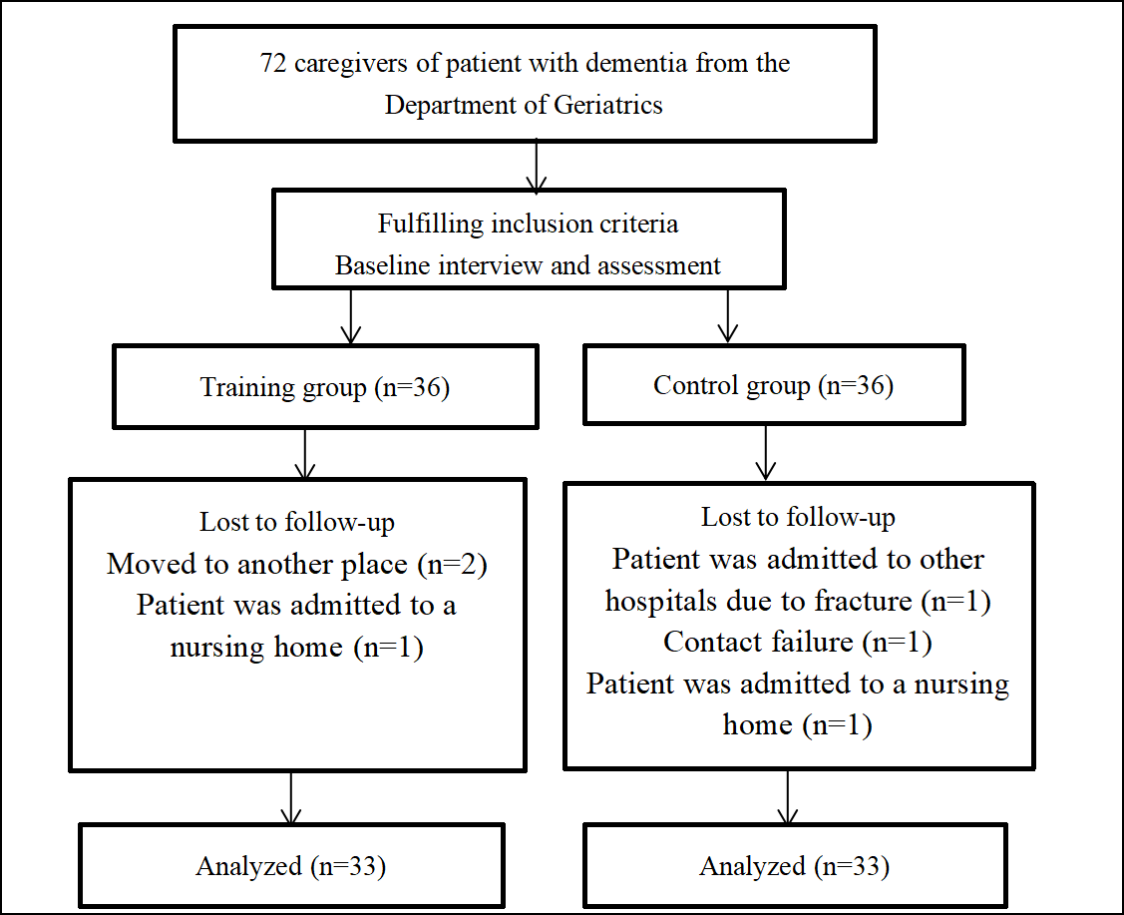
Flowchart of study participants.

In the follow-up period, 3 patients in the intervention group and the control group were lost to follow-up, respectively. In the intervention group, 2 caregivers accompanied the patients with dementia when they relocated to a different city, and another caregiver returned to her hometown after the patient with dementia was admitted to a nursing home. In the control group, one caregiver of a patient with dementia accompanied the patient for emergency hospital admission due to a fracture, another caregiver failed to continue contact despite repeated appointments, and one caregiver accompanied the patient to a nursing home (Figure 1).

### Sample Size Calculation

The experiment was conducted as a parallel-group randomized controlled trial study design, and repeated measures were used. Sample size calculation was based on the primary outcome of the Chinese version of the Zarit Burden Interview (CZBI) at 6 months after baseline. The detection power was set at 90%, and α (2-tailed) was set at .05. Preliminary experiments showed that the CZBI of dementia family caregivers was 21.55, with an SD of 9.89, and an auto-correlation between adjacent measurements on the same individual was 0.70. By providing web-based knowledge and skills training through the internet, the preliminary trial showed that the average CZBI had decreased by 12.5. Using PASS software (version 16.0; National Council on Social Studies), the minimum sample size for each group was identified as 27. Considering a potential 10% loss of samples during the study, the final sample size was determined to be 36 individuals per group.

### Intervention

The platform aims to provide opportunities for support, education, and sharing experiences among users across Zhejiang province. In order to provide users with convenient access to the course at any time and place, the platform was designed to operate on any internet-enabled device. In the intervention group, we instructed the informal caregivers of persons with dementia to log in to the platform web page and taught them about the knowledge and skills relevant to home care via the internet. The content on the platform was divided into 5 different themes, which were: computer cognitive training, language training, reality-oriented therapy, daily life rehabilitation, and care skills training. Computer cognitive training includes concentration (picture-text matching), memory (vocabulary memorization), calculation (simulated shopping), and reaction training (fruit picking). Each training time was 20 minutes and was conducted 3 times a week. Language training aims to improve communication by focusing on vocalization, recognition, and the application of words. Reality orientation therapy focuses on presenting patients with facts about the time, date, and current environment. The primary objective for daily life rehabilitation is to increase self-care ability. The 4 themes (cognitive training, language training, reality-oriented therapy, and daily life rehabilitation) have been evaluated in a prior study with pending results and this paper focuses mainly on the fifth theme. The latter theme consisted of 18 lessons (270 min) aimed at improving caregiver care skills (Table 1). In order to encourage participants to complete the web-based course training, the web-based active screen time was calculated. Once participants completed at least 80% of the training sessions (216 min), they would be sent a book (“Long-term Care for Dementia”).

The control-comparison group would receive an educational booklet on caring for patients with dementia provided by the research team. In addition to this education, the control group would also receive face-to-face follow-up guidance and have the option to receive the same intervention after 6 months. The research team’s helpline would also be available 24 hours a day to the control group for caregiver assistance.

**Table 1.** Overview of intervention modules.

Theme	Curriculum content
Recognition of dementia	The concept of dementia The overview of dementia care Types and causes of dementia
Physical care for dementia	Nutritional care for dementia Excretory care for dementia Nonpharmacologic interventions for sleep disorders in dementia BPSD^a^ and response strategies
Physical activity maintenance	Accompany shopping Accompany outdoor activities Preventing and delaying the onset of dementia BPSD syndrome
Good memories: cognitive facilitation of care	Memory training: vocabulary memorization Computational skill training: calculation (simulated shopping) Manual activities: reaction training (fruit picking) Music therapy: this module includes 100 Chinese revolutionary and nostalgic songs, and encourages family caregivers to customize personalized music playlists for the elderly, put them in the proper order, and play them at specific times or activities, such as when they wake up in the morning, at lunchtime, or during evening relaxation time Spatial orientation training: the platform displays the date, day, weather, season, and holiday. The platform provides prompts to guide the patient in answering questions related to the direction of time Language training: identifying and describing objects from pictures, finding the correct word, and idiomatic solitaire
Safe harbor: health promotion care	Safety risks and prevention in home care for older people with dementia: improving the living environment; arranging for special care; setting up reminders such as “Watch out for slipping” and “CAUTION//Hot Water”; avoiding the use of dangerous items such as knives and matches; conducting regular checkups Emergency response plan for patients with dementia with sudden incidents (stray, fall, and choking) Sharing family care experience of dementia

a BPSD: behavioral and psychological symptoms of dementia.

### Measurement Instruments

Due to the in-person meeting restrictions imposed by the COVID-19 pandemic, caregivers participated in two 10-minute web-based self-assessed surveys through Questionnaire Star at 3 and 6 months. The primary outcome measures were the Neuropsychiatric Inventory Questionnaire (NPI-Q), CZBI, and the Sense of Competence in Dementia Care Staff Scale (SCIDS).

The NPI-Q [16] is an observer rating scale that was used to evaluate participants’ neuropsychiatric symptoms across 12 different areas, including delusions, hallucinations, agitation/aggression, depression/dysphoria, anxiety, elation/euphoria, apathy/indifference, disinhibition, irritability/emotional lability, aberrant motor behaviors, nighttime behavioral disturbances, and appetite/eating disturbances. Each symptom was evaluated with a basic screening question (to which participants responded). If a positive answer is given to the screening question, a more detailed exploration of specific areas will be conducted. The neurobehavioral manifestations within a domain are collectively rated by caregivers based on frequency (1 to 4) and severity (1 to 3), resulting in a composite domain score (frequency × severity), with higher scores indicating greater severity of symptoms. The total NPI-Q score is defined as the sum of scores from 12 symptom evaluations, with a maximum score of 144. The Cronbach α coefficient of the NPI-Q was .82, and the test-retest coefficients ranged from .66 to .98 (*P*<.001) [17].

The caregiver burden was measured using the CZBI [18]. CZBI consists of 22 items that require a Likert-type response ranging from 0 (never) to 4 (almost always), with a total score ranging from 0 to 88. A higher score indicates an increased caregiver burden. The internal consistency value, established by Cronbach α coefficient, was .89 and the intraclass correlation coefficient for test-retest reliability of the total score was 0.88.

SCIDS is designed to evaluate the level of competency among caregivers in providing care for individuals with dementia [19]. The scale consists of 17 items across 4 subscales: professionalism, relationship-building, care challenges, and sustaining personhood. SCIDS has acceptable to good internal consistency (Cronbach *α*=.91) and moderate to substantial test-retest reliability (0.74). The total score on the scale ranges from 17 to 68 points, with higher scores indicating that staff members have a better awareness of dementia care abilities.

### Statistical Analysis

Outliers were identified using boxplots, and data distribution was assessed by the Shapiro-Wilk test. Data were tested for normal distribution or variance homogeneity using appropriate tests before statistical analysis with SPSS statistical software (version 20.0; IBM Corp). Continuous variables that followed a normal distribution were expressed as mean and SD and were compared using an independent *t* test between the 2 groups. Categorical variables were expressed as frequencies and proportions and compared using a chi-square test between groups. In repeated measures analysis of variance (ANOVA), when the assumption of sphericity was violated (evaluated using the Mauchly test), the degrees of freedom value for testing the *F-*ratio was adjusted using the Greenhouse-Geisser correction. If there was no interaction effect between time and treatment factors in the repeated measures ANOVA results, the main effects test was used to evaluate the treatment effect. If there was an interaction effect, separate analyses were performed: the within-group effect was evaluated using a one-way repeated measures ANOVA, and the between-group effect was evaluated using a multivariate ANOVA. Bonferroni correction was used for posthoc multiple pairwise comparisons.

## Results

### Sample Characteristics

The patients in the control and intervention groups were comparable in terms of their baseline data such as sex, education level, marital status, age, BMI, number of children, number of diseases, and number of long-term medications (Table 2). In this study, caregivers averaged 57.74 years old (SD 3.93; range 48-66) and the majority (57/66, 86%) were female. Caregivers were most frequently the patients’ distant relatives from the countryside (42/66, 64%) and children (13/66, 20%); the remainder were spouses (11, 16%). Table 2 presents the demographic characteristics of the sample. Among the study groups, demographic and clinical characteristics did not differ significantly.

**Table 2.** Sample characteristics.

Category		Intervention group (n=33)	Control group (n=33)
**Caregiver age (years), mean (SD); range**		58.79 (4.08); 48‐66	56.70 (3.47); 50‐64
**Sex, n (%)**			
	Male	3 (9)	5 (16)
	Female	30 (91)	27 (84)
**Caregiver relationship, n (%)**			
	Spouse	5 (15)	6 (18)
	Distant relatives from the countryside	22 (67)	20 (61)
	Son or daughter (-in law)	6 (18)	7 (21)
**Education level, n (%)**			
	Illiterate or primary school	21 (64)	19 (58)
	Junior school	5 (15)	4 (12)
	High school or above	7 (21)	10 (30)
**Outcome variables at baseline, mean (SD)**			
	NPI-Q^a^	5.97 (6.14)	4.45 (5.43)
	CZBI^b^	24.48 (10.77)	19.85 (11.55)
	SCIDS^c^	59.64 (5.63)	59.76 (7.36)

a NPI-Q: Neuropsychiatric Inventory Questionnaire.

b CZBI: Chinese version of the Zarit Burden Interview.

c SCIDS: Sense of Competence in Dementia Care Staff Scale.

### Findings

According to the results of repeated measures ANOVA, as shown in Table 3, it was found that intervention and time had significant statistical effects on the NPI-Q, CZBI, and SCIDS (*P*<.05), and the significant interaction effects of group × time in primary outcome indicators were also found (*P*<.05). Since there were significant differences (group × time effect) in the primary outcome measure, analyses of the individual effects of intervention and time were performed. To verify the significance of intervention effects, multiple comparisons were subjected to the Bonferroni correction for adjustment. The comparison of various data between the intervention group and the control group at different time points is shown in Table 4. After 6 months of intervention, the NPI-Q of caregivers in the intervention group was significantly lower than that of in the control group (*t*=−3.020, *P*=.004); at 3 and 6 months after intervention, the care burden (CZBI) of caregivers in the intervention group was significantly lower than that of in the control group (*t*_3month_=−2.939, *P*=.005; *t*_6month_=−7.858, *P*<.001). The caregiving ability (SCIDS) of caregivers was significantly higher than that of the control group (*t*_3month_=6.138, *P*<.001; *t*_6month_=12.16, *P*<.001).

Using the Sidak method to conduct multiple comparisons for the individual effects of time on the primary outcome measure (NPI-Q, CZBI, and SCIDS), the results are shown in Table 5. In the intervention group, the NPI-Q score showed a decrease over time (mean difference_T2-T1_=−1.39, mean difference_T3-T1_=−2.79), while the comparison group showed a significant increase at the 3- and 6-month follow-up compared with baseline, with statistically significant differences (mean difference_T2-T1_=1.46, mean difference_T3-T1_=3.64). According to the research results, the severity of NPI-Q in patients with dementia tends to worsen over time. However, this study showed that caregiver knowledge and skills training through an information platform significantly delayed the progression of NPI-Q symptoms in patients with dementia, indicating the effectiveness of this training method.

The intervention group’s CZBI scores showed a significant decrease over time (mean difference_T2-T1_=−7.70, mean difference_T3-T1_=−13.52), while the control group experienced a significant increase (mean difference_T2-T1_=4.49, mean difference_T3-T1_=10.45). The study revealed that internet-based supportive interventions providing dementia caregivers with knowledge and skills training led to a significant reduction in caregiving burden and were proved to be highly effective.

In terms of caregiving ability (SCIDS), the performance of the intervention group’s caregivers significantly improved in the 3- and 6-month follow-ups, with statistically significant differences compared with before intervention (mean difference_T2-T1_=5.79, mean difference_T3-T1_=12.24). Conversely, the caregiving ability of the control group’s caregivers declined during the same period (mean difference_T2-T1_=−6.70, mean difference_T3-T1_=−8.64). This result indicated that providing knowledge and skills training to informal caregivers of persons with dementia through information technology platforms can effectively enhance their caregiving skills.

**Table 3.** Comparison of 3 scores between 2 randomly assigned groups with key outcomes that vary over time.

Measure		NPI-Q^a^	CZBI^b^	SCIDS^c^
**Baseline**				
	Intervention group	5.97 (6.14)	24.48 (10.77)	59.64 (5.63)
	Control group	4.45 (5.43)	19.85 (11.55)	59.76 (7.36)
**3 months after intervention**				
	Intervention group	4.58 (4.95)	16.79 (8.05)	65.42 (6.15)
	Control group	5.91 (6.44)	24.33 (12.35)	52.06 (10.89)
**6 months after intervention**				
	Intervention group	3.18 (3.81)	10.97 (5.43)	71.88 (4.78)
	Control group	8.09 (8.52)	30.30 (13.05)	50.12 (9.09)
***F***_**intergroup effect**_		1.282	9.009	61.86
	*P* value	.26	.004	<.001
	Partial η^2^	0.02	0.123	0.491
***F***_**time effect**_		0.516	3.469	3.205
	*P* value	.58	.05	.05
	Partial η^2^	0.008	0.051	0.048
***F***_**interaction effect**_		23.856	151.706	63.03
	*P* value	<.001	<.001	<.001
	Partial η^2^	0.272	0.703	0.492

a NPI-Q: Neuropsychiatric Inventory Questionnaire.

b CZBI: Chinese version of the Zarit Burden Interview.

c SCIDS: Sense of Competence in Dementia Care Staff Scale.

**Table 4.** Comparison of 3 scores between the 2 groups at different time points.

Scale		Intervention group, mean (SD)	Control group, mean (SD)	*t*	*P* value
**NPI-Q** ^**a**^					
	Baseline	5.97 (6.14)	4.45 (5.43)	1.062	.29
	3 months	4.58 (4.95)	5.91 (6.44)	−0.943	.35
	6 months	3.18 (3.81)	8.09 (8.52)	−3.020	.004
**CZBI** ^**b**^					
	Baseline	24.48 (10.77)	19.85 (11.55)	1.687	.10
	3 months	16.79 (8.05)	24.33 (12.35)	−2.939	.005
	6 months	10.97 (5.43)	30.30 (13.05)	−7.858	<.001
**SCIDS** ^**c**^					
	Baseline	59.64 (5.63)	58.76 (7.36)	0.545	.59
	3 months	65.42 (6.15)	52.06 (10.89)	6.138	<.001
	6 months	71.88 (4.78)	50.12 (9.10)	12.160	<.001

a NPI-Q: Neuropsychiatric Inventory Questionnaire.

b CZBI: Chinese version of the Zarit Burden Interview.

c SCIDS: Sense of Competence in Dementia Care Staff Scale.

**Table 5.** Time for multiple comparisons of the individual effects of the Neuropsychiatric Inventory Questionnaire (NPI-Q), Chinese version of the Zarit Burden Interview (CZBI), and Sense of Competence in Dementia Care Staff Scale (SCIDS).

Measure		T2-T1		T3-T1		T3-T2	
		Mean difference (95% CI)	Adjusted *P* value	Mean difference (95% CI)	Adjusted *P* value	Mean difference (95% CI)	Adjusted *P* value
**Intervention group**							
	NPI-Q	−1.39 (−2.99 to 0.20)	.11	−2.79 (−4.38 to −1.19)	<.001	−1.39 (−2.99 to 0.20)	.11
	CZBI	−7.70 (−10.05 to −5.34)	<.001	−13.52 (−15.87 to −11.16)	<.001	−5.82 (−8.17 to −3.46)	<.001
	SCIDS	5.79 (2.56 to 9.02)	<.001	12.24 (9.02 to 15.47)	<.001	6.46 (3.23 to 9.68)	<.001
**Control group**							
	NPI-Q	1.46 (−0.14 to 3.05)	.09	3.64 (2.04 to 5.23)	<.001	2.18 (0.59 to 3.78)	.004
	CZBI	4.49 (2.13 to 6.84)	<.001	10.45 (8.10 to 12.81)	<.001	5.97 (3.62 to 8.32)	<.001
	SCIDS	−6.70 (−9.92 to −3.47)	<.001	−8.64 (−11.86 to −5.41)	<.001	−1.94 (−5.17 to 1.29)	.38

## Discussion

### Principal Findings

The restrictions of the COVID-19 pandemic have had profound effects on patients with dementia and their caregivers. This study shows that internet-based support and education have a positive impact on family caregivers. The intervention can reduce the NPI-Q scores of patients with dementia, enhance the dementia care skills of informal caregivers, and ease the care burden for caregivers. With the progression of dementia, caregivers not only have to deal with many behavioral, daily living, and safety issues but also lack the professional knowledge and skills to address diverse caregiving challenges. This not only causes them to experience anxiety and depression but also brings care and financial burdens to their families and society. Web-based interventions based on internet technology are highly flexible and suitable for family caregivers, as their schedules are largely consumed by caregiving and they may not have time to attend in-person learning sessions (due to travel and time constraints).

Patients diagnosed with dementia often struggle with a gradual deterioration of emotional regulation and an increased likelihood of displaying behavioral and psychological symptoms. This is closely linked to the decline in cognitive function associated with the condition. Research on caregiver burden indicated that BPSD are strongly correlated with caregiver burden [20]. The prevalence of dementia in Chinese society is expected to increase in the next 40 years. Family members remain the primary caregivers and bear a heavy burden. BPSD not only has a significant impact on the health and quality of life of patients but also brings enormous physical and psychological burdens to caregivers. In this study, the intervention group had significantly lower NPI-Q scores than the control group after 6 months of intervention. The results indicated that internet-based supportive interventions for dementia caregivers can effectively improve BPSD symptoms in patients with dementia. This result is consistent with those of previous studies [21,22]. BPSD symptoms are a prevalent issue among older individuals with dementia. These symptoms often occur repeatedly and can present at varying stages of the disease. Previous research [23] has demonstrated that the emergence of BPSD symptoms is associated with an increased risk of accelerated progression of dementia, reduced cognitive function, and a quicker decline in cognitive abilities in individuals with dementia. Our research has to some extent reduced the incidence and severity of behavioral and emotional symptoms. The meta-analysis results of Leng et al [24] also show that internet-based supportive interventions have potential benefits for the neurological and psychiatric symptoms of patients with dementia.

The research results showed that after 6 months of intervention, the total score of caregiving ability for the intervention group caregivers was significantly higher than that of the control group (*P*<.05), indicating that web-based supportive intervention for dementia informal caregivers can significantly improve their caregiving ability. Providing care for patients with dementia is an incredibly difficult task, and caregivers may struggle with a lack of essential resources, including knowledge, skills, and social support. In this context, the development of knowledge and skills to support family caregivers is essential. The web-based caregiver training intervention plan for dementia caregivers in this project covers the basic knowledge that caregivers should possess. It systematically explains the causes, clinical manifestations, and progression of dementia, promoting caregivers’ understanding. Mastering knowledge is a prerequisite for improving skill levels [25]. In caring for patients with dementia, knowing about the disease can help caregivers better care for patients. Only when caregivers have a correct understanding of the illness can they truly empathize with patients and provide them with appropriate care. This study adopted web-based training to enhance caregivers’ caregiving skills from 4 aspects: knowledge, skills, self-emotional management, interpersonal relationships, and resource use. In terms of intervention form, web-based knowledge and skills training is more flexible in terms of time, allowing caregivers to choose their free time to increase their participation. The result is similar to the study by Teles et al [26].

After comparing the CZBI scores of the 2 groups of caregivers at different time points, we concluded that within 6 months after the intervention, the CZBI scores of caregivers in the intervention group were significantly lower than those in the control group (*P*<.05). The findings demonstrated that the internet-based supportive intervention had a significant impact on alleviating the burden of care for dementia. This result is consistent with those of 2 studies [26,27]. The meta-analysis carried out by Egan et al [28] and the systematic review conducted by Pleasant et al [29] have also demonstrated web-based training programs’ supportive intervention for informal caregivers of persons with dementia were highly effective in reducing their burden. When informal caregivers have mastered basic care knowledge and skills, their confidence in caring for patients is enhanced, uncertainty is reduced, and understanding of patients is improved. This knowledge and skills can reduce the burden on caregivers and enable them to care for patients more effectively. Using home care resources could help maintain health status, minimize symptom relief, and reduce avoidable hospitalizations [30]. This study offers a solution to the challenges faced by informal caregivers by providing them with training through web-based programs. They gain knowledge and skills that allow them to adjust their caregiving schedules flexibly to accommodate the needs of individuals with dementia. This approach not only empowered informal caregivers but also optimized the use of home care resources, making care provision more effective.

### Limitations

This study has limitations. First, due to time constraints, the effects of the intervention program in this study were evaluated for only 6 months, and long-term follow-up will be conducted in the future. Second, since manpower, funding, and time constraints prevented long-term research, this study was conducted with a small sample size only. Third, we measured outcomes and exposures using self-reported questionnaires, so reporting errors was possible. Despite these limitations, our plan is still noteworthy because it is one of the few web-based interventions that have a significant impact on fostering positive emotions towards caregiving and reducing the burden on informal caregivers of persons with patients with dementia. Bastoni et al’s [31] research has found that monitoring devices are rapidly developing and are seen as promising technologies. This includes monitoring health and safety in homes, as well as providing outdoor location identification for patients with dementia. In the future, we will conduct in-depth research on integrating wearable devices and other mobile information collection terminals into this information platform to achieve real-time monitoring of the health status of patients with dementia.

### Conclusions

The long-term care system in China predominantly relies on informal family care. Family caregivers are the mainstay of elderly care at present in China, and their caregiving capacity directly affects the quality of family care. The lack of caregiver competence affects the quality of care because family caregivers lack basic knowledge and relevant skills in caregiving. The Chinese eldercare system relies heavily on informal care provided by family members due to filial piety. China has many patients with dementia due to its huge population base. Family caregivers are the mainstay of elderly care in China currently [32], and their ability to provide care directly affects the quality of home-based care. Caregivers for patients with dementia often face the reality of inadequate caregiving skills and a desire for support. As a family caregiver, one needs to possess a variety of knowledge and skills. This comprises an understanding of the ailment in question, being adept at solving problems creatively, and possessing techniques for maintaining psychological well-being. Informal caregivers play a crucial role in the care of individuals with dementia. Therefore, it is imperative to have interventions in place that not only support them but also alleviate their burden. Major technological advancements should be leveraged to optimize the time and effectiveness of the dementia care workforce [33]. As a cost-effective, convenient, and accessible intervention, web-based solutions have emerged to support informal caregivers. The advantage of web-based support training lies in breaking the limitations of time and space and providing a feasible solution for the popularization of support services. During the COVID-19 pandemic, our research project provided digital resources through web-based support and training to informal caregivers of persons with dementia, which improved the caregivers’ skills and alleviated their burden.

## Supplementary material

10.2196/50847 Checklist 1 CONSORT-EHEALTH (V 1.6.1).

## Abbreviations

ANOVA: analysis of variance
BPSD: behavioral and psychological symptoms of dementia
CZBI: Chinese version of the Zarit Burden Interview
NPI-Q: Neuropsychiatric Inventory Questionnaire
SCIDS: Sense of Competence in Dementia Care Staff Scale
