# Correction: Internet-Based Supportive Interventions for Family Caregivers of People With Dementia: Randomized Controlled Trial

**DOI:** 10.2196/69493

**Published:** 2025-01-29

**Authors:** Yanhong Xie, Shanshan Shen, Caixia Liu, Hong Hong, Huilan Guan, Jingmei Zhang, Wanqi Yu

**Affiliations:** 1 Geriatrics Department, Zhejiang Hospital, Hangzhou, China; 2 Nursing Department, Zhejiang Hospital, Hangzhou, China; 3 General Surgery, Zhejiang Hospital, Hangzhou, Zhejiang Province, China; 4 The Medical Record Statistics Department, Zhejiang Hospital, Hangzhou, China

In “Internet-Based Supportive Interventions for Family Caregivers of People With Dementia: Randomized Controlled Trial” (JMIR Aging. 2024 Oct 4:7:e50847. doi: 10.2196/50847) the authors made three corrections.

The following funding information has been added to the end of the “Acknowledgements” section:

*This work was supported by grants from the National Key Research and Development Program of China (program number: 2019YFE0113100), the Zhejiang Provincial Medicine and Health Technology Project (grant number: 2019KY003), and the Zhejiang Hospital 3060 Excellent Young Talents Training Project*.

Additionally, the authors would like to specify equal contribution betweenYanhong Xie and Shanshan Shen as co-first authors, and Hong Hong and Caixia Liu as co-corresponding authors. The latter will be specified at the beginning of the “Acknowledgements” section as follows:

*CL is the co-corresponding author on this work, and can be reached at the following email address: zjyyhlb2007@126.com.*

Finally, the following ORCID has been added to author Hong Hong:

*0009-0001-1372-1670*

The corrections will appear in the online version of the paper on the JMIR Publications website on January 29, 2025, together with the publication of this correction notice. Because this was made after submission to PubMed, PubMed Central, and other full-text repositories, the corrected article has also been resubmitted to those repositories.

